# Development and regulation of chloride homeostasis in the central nervous system

**DOI:** 10.3389/fncel.2015.00371

**Published:** 2015-09-24

**Authors:** Miho Watanabe, Atsuo Fukuda

**Affiliations:** Department of Neurophysiology, Hamamatsu University School of MedicineHamamatsu, Japan

**Keywords:** KCC2, NKCC1, GABA, glycine, taurine, WNK, PKC, BDNF

## Abstract

γ-Aminobutyric acid (GABA) is the main inhibitory neurotransmitter of the mature central nervous system (CNS). The developmental switch of GABAergic transmission from excitation to inhibition is induced by changes in Cl^−^ gradients, which are generated by cation-Cl^−^ co-transporters. An accumulation of Cl^−^ by the Na^+^-K^+^-2Cl^−^ co-transporter (NKCC1) increases the intracellular Cl^−^ concentration ([Cl^−^]_i_) such that GABA depolarizes neuronal precursors and immature neurons. The subsequent ontogenetic switch, i.e., upregulation of the Cl^−^-extruder KCC2, which is a neuron-specific K^+^-Cl^−^ co-transporter, with or without downregulation of NKCC1, results in low [Cl^−^]_i_ levels and the hyperpolarizing action of GABA in mature neurons. Development of Cl^−^ homeostasis depends on developmental changes in NKCC1 and KCC2 expression. Generally, developmental shifts (decreases) in [Cl^−^]_i_ parallel the maturation of the nervous system, e.g., early in the spinal cord, hypothalamus and thalamus, followed by the limbic system, and last in the neocortex. There are several regulators of KCC2 and/or NKCC1 expression, including brain-derived neurotrophic factor (BDNF), insulin-like growth factor (IGF), and cystic fibrosis transmembrane conductance regulator (CFTR). Therefore, regionally different expression of these regulators may also contribute to the regional developmental shifts of Cl^−^ homeostasis. KCC2 and NKCC1 functions are also regulated by phosphorylation by enzymes such as PKC, Src-family tyrosine kinases, and WNK1–4 and their downstream effectors STE20/SPS1-related proline/alanine-rich kinase (SPAK)-oxidative stress responsive kinase-1 (OSR1). In addition, activation of these kinases is modulated by humoral factors such as estrogen and taurine. Because these transporters use the electrochemical driving force of Na^+^ and K^+^ ions, topographical interaction with the Na^+^-K^+^ ATPase and its modulators such as creatine kinase (CK) should modulate functions of Cl^−^ transporters. Therefore, regional developmental regulation of these regulators and modulators of Cl^−^ transporters may also play a pivotal role in the development of Cl^−^ homeostasis.

## Introduction

γ-Aminobutyric acid (GABA), which hyperpolarizes membrane potential and dampens neuronal excitability, is the main inhibitory neurotransmitter of the central nervous system (CNS). GABA opens Cl^−^-permeable GABA_A_ receptor-channels (GABA_A_R) and via Cl^−^ influxes down the electrochemical gradient, hyperpolarizes the membrane potential, which results in inhibitory actions of GABA. However, when intracellular Cl^−^ concentrations ([Cl^−^]_i_) are high, the equilibrium potential for Cl^−^ (E_Cl_) can be positive compared to the resting membrane potential. In this situation, GABA can depolarize the membrane potential beyond the threshold of action potential generation, indicating that it is an excitatory neurotransmitter.

Low [Cl^−^]_i_ allows adult neurons to hyperpolarize in response to GABA_A_R activation by GABA. Immature neurons, in contrast, exhibit high [Cl^−^]_i_. Therefore, GABA induces depolarization and can be excitatory. Developmental GABAergic excitation-inhibition switches are induced by changes in Cl^−^ gradients, which are generated by cation-Cl^−^ co-transporters. The Na^+^-K^+^-2Cl^−^ co-transporter (NKCC1)-mediated accumulation of Cl^−^ results in increased intracellular Cl^−^ levels in neuronal precursors and immature neurons. This allows for depolarizing (excitatory) actions of GABA in the developing brain (Alvarez-Leefmans et al., 2001; Ben-Ari, 2002; Payne et al., 2003; Gamba, 2005; Ben-Ari et al., 2007; Blaesse et al., 2009). The subsequent ontogenetic switch, i.e., the upregulation of the Cl^−^-extruder KCC2, which is a neuron-specific K^+^-Cl^−^ co-transporter, and the concomitant downregulation of NKCC1, results in low [Cl^−^]_i_ levels and hyperpolarization by GABA in rat neocortex (Yamada et al., 2004). Indeed, NKCC1 is highly expressed in the neonatal rat cortex, whereas KCC2 is expressed at only 5–15% of adult expression levels (Dzhala et al., 2005).

Because Kahle et al. (2013), Kaila et al. (2014) and Medina et al. (2014) have recently extensively reviewed regulatory factors of KCC2, including its cytoskeletal role in the dendritic spine, we focus here on the development of Cl^−^ transporters together with their upstream regulators, which are directly involved in the ontogenesis of the Cl^−^ homeostasis.

## Cation-Cl^−^ Co-Transporters

**Figure 1:**
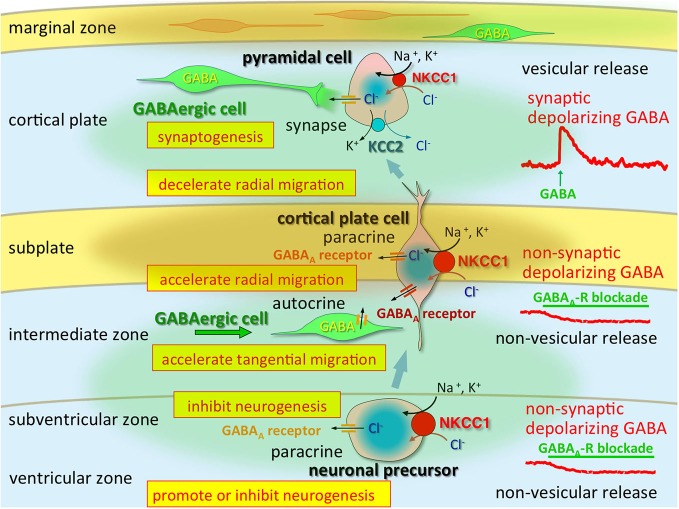
**Multi-modal actions of tonic modulation of the GABA_A_ receptor during corticogenesis.** In the ventricular zone (VZ) and subventricular zone (SVZ), ambient GABA affects neurogenesis. Post-mitotic neurons migrating to the cortical plate are tonically depolarized by non-synaptic and non-vesicular ambient GABA, released from tangentially migrating GABA neurons prior to forming synapses, and changing their migratory pace. GABA also acts in an autocrine manner to accelerate tangential migration. Vesicular release of GABA, which remains depolarizing, may contribute to synapse formation. Immediately after establishment of GABAergic synapses (and prior to hyperpolarization), GABA acts as an excitatory neurotransmitter due to high [Cl^−^]_i_, predominantly resulting from high NKCC1 expression, while the KCC2 expression level remains low. Green circle: ambient GABA. Brown circle: ambient taurine. Adapted from Egawa and Fukuda (2013).

Altered GABAergic functions that occur during early brain development are induced by changes in Cl^−^ homeostasis (Shimizu-Okabe et al., 2002; Yamada et al., 2004) and play important roles in neocortical development by modulating several events of corticogenesis (Egawa and Fukuda, 2013; Luhmann et al., 2015; Figure 1). KCC2 mRNA is not detectable in neuronal precursors or in migrating cells but is observed in differentiated neurons (Li et al., 2002; Wang et al., 2002; Stein et al., 2004). In contrast, NKCC1 is expressed strongly throughout the neuroepithelium, where KCC2 is never observed (Li et al., 2002; Wang et al., 2002). This demonstrates the striking contrast in the natures of these co-transporters.

In immature neurons with low expression of KCC2, GABAergic excitation is dependent on high NKCC1 activity. Achilles et al. (2007) revealed steady-state [Cl^−^]_i_ at 30 mM, which was reduced to values close to passive distribution by bumetanide, a NKCC1 inhibitor, and by Na^+^-free solutions, which suggests a role for NKCC1 in increasing [Cl^−^]_i_. Indeed, NKCC1 is strongly expressed in these cells, and active Cl^−^ uptake occurs with a velocity of 50 μMS^-1^, such that the NKCC1-mediated Cl^−^ uptake is capable of maintaining excitatory GABAergic membrane responses in Cajal-Retzius cells (Achilles et al., 2007). Therefore, if NKCC1 is abundant and slow Cl^−^ uptake mediated by this transporter is active, it would be sufficient to maintain the high [Cl^−^]_i_ that is essential for excitatory GABA_A_R responses.

**Figure 2:**
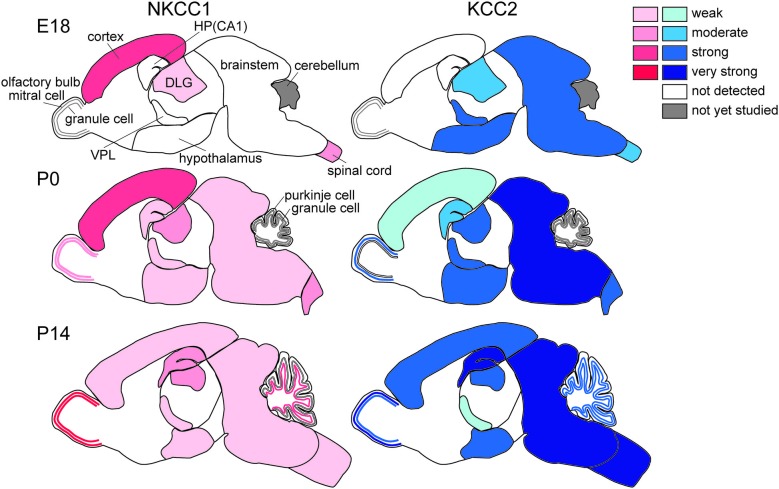
**Differential development of NKCC1 (red) and KCC2 (blue) expression in the brain.** HP, hippocampus; DLG, dorsal lateral geniculate nucleus; VPL, ventral posterior thalamic nucleus (Mikawa et al., 2002; Shimizu-Okabe et al., 2002; Wang et al., 2002, 2005; Ikeda et al., 2003).

The regional differences in Cl^−^ homeostasis during development are apparent (Figure 2). In general, evolutionarily older brain structures that develop earlier show more advanced maturation of Cl^−^ homeostasis, as described in more detail below. The neurogenesis of the rat brain extends from embryonic day (E)12 to postnatal day (P)19 (Bayer and Altman, 1995). The timetables for neurogenesis in the rat brain show a highly sequential pattern among different neuronal populations (Bayer and Altman, 1995). The oldest neurons exist in the spinal cord and medulla and are born mainly on E12–E13. Neurons in the thalamus, hypothalamus and amygdala are mainly generated during E13–E16. In the neocortex, most neurons originate during E16–E18. In the hippocampus, pyramidal neurons arise during E17–E19 and dentate granule cells are born after birth. KCC2 mRNA expression follows this same sequence. On E18, strong KCC2 mRNA expression was observed to be already present in the thalamus, hypothalamus, and amygdala. In contrast, in the neocortex and hippocampus, the earliest strong KCC2 mRNA expression is observed on P15. Thus, the expression profile of KCC2 mRNA in the developing rat brain is well correlated with the sequential maturation of neurons (Li et al., 2002; Wang et al., 2002; Stein et al., 2004). Generally, the ontogeny of KCC2 mRNA in mouse brain is similar to that in rat brain, but the timing includes delays of 2 days in rat embryos (Li et al., 2002).

The spinal cord and brainstem show the earliest development of Cl^−^ homeostasis. Expression of KCC2 mRNA starts as early as E10.5 (in mice), at which time it is restricted to the spinal cord and the brainstem. KCC2 transcripts are found in developing motoneurons in the ventral horn of the spinal cord and the medulla as early as E12.5 (Hübner et al., 2001) and in sensory nuclei at later stages, E15.5 in mice, and the extent of its expression increases during embryonic development (Stein et al., 2004). Regardless of this early expression of KCC2 in the spinal cord, GABA_A_R- and glycine receptor-mediated Cl^−^ currents are depolarizing. Delpy et al. (2008) showed that in mouse embryonic spinal cord, both KCC2 and NKCC1 are expressed and functional early in development (E11.5–E13.5), when GABA_A_R activation evokes excitatory action. After E15.5, NKCC1 becomes inactive and sparse in motoneurons while KCC2 remains functional and provides more negative E_Cl_, so that GABA and glycine act as inhibitory neurotransmitters in the majority of spinal motoneurons in the mouse at E17.5, (Branchereau et al., 2002) and at E17–E19 in the rat (Wu et al., 1992; Sibilla and Ballerini, 2009). This switch may correspond to the period when locomotor networks start to generate alternating flexor and extensor motor activities concomitantly to the network expression of left-right alternation, which indicates the presence of functional network inhibition (Delpy et al., 2008; Sibilla and Ballerini, 2009). In rats, Stil et al. (2009) showed that the switch from depolarizing to hyperpolarizing inhibitory postsynaptic potentials (IPSPs) occurs at P4–P5 in the ventral horn of rat spinal cord, in agreement with the evidence for increases in KCC2 expression together with decreases in NKCC1 expression during postnatal life. In any case, in the spinal cord, KCC2 and NKCC1 are expressed throughout embryonic development, but NKCC1 becomes inefficient during maturation (Delpy et al., 2008). Similarly, in the mouse pre-Bötzinger complex, which is a brainstem respiratory structure driving rhythmic activity of the hypoglossal motoneurons, the action of GABA switches from depolarizing to hyperpolarizing within the first postnatal week, usually between P2 and P4 (Ritter and Zhang, 2000).

KCC2 mRNA is strongly expressed in the hypothalamus soon after its differentiation in rodents, as early as E14.5, and continues to be expressed postnatally (Li et al., 2002; Mikawa et al., 2002; Wang et al., 2002; Stein et al., 2004), whereas NKCC1 expression is not obvious in embryos and is weakly expressed postnatally (Li et al., 2002; Wang et al., 2002). These findings suggest that Cl^−^ homeostasis in the developing hypothalamus is already of the mature type, i.e., GABA has inhibitory effects. However, electrophysiological experiments indicate that GABA acts in an excitatory manner at early stages after birth (Gao and van den Pol, 2001). This discrepancy may be explained by low KCC2 protein levels and/or cell-type-specific (Watanabe et al., 2014) and/or sex-specific differences in the expression of KCC2 and NKCC1 (more KCC2 and less NKCC1 in females; Perrot-Sinal et al., 2007). Posttranslational mechanisms such as phosphorylation of NKCC1 and KCC2 via STE20/SPS1-related proline/alanine-rich kinase (SPAK)/OSR-1 (Kahle et al., 2013) might also be involved, because these mechanisms may be modulated by humoral factors such as estrogen (Nugent et al., 2012) and taurine (Inoue et al., 2012).

As in the hypothalamus, the embryonic thalamus, the largest part of the developing diencephalon, already expresses KCC2 mRNA at E12 when it begins to form. At E15.5, KCC2 is predominantly present in the dorsolateral nuclei of the thalamus, where neuronal differentiation of thalamic neurons originates (Niimi et al., 1961). In contrast, the undifferentiated dorsomedial parts do not express KCC2 but begin to do so later, at E18.5. KCC2 mRNA expression is also found in the ventral thalamus and ventral lateral geniculate nucleus as early as E14.5 both in rats and mice (Li et al., 2002; Wang et al., 2002). Interestingly, KCC2 mRNA expression declines after birth in the ventral posterior thalamic nucleus: in the rat, KCC2 mRNA expression is observed strongly on P1 but only weakly on P15 and P40 (Wang et al., 2002). Also, interestingly, NKCC1 is not expressed in the thalamus during the embryonic period but is stably expressed from soon after birth to adulthood (Wang et al., 2002). This indicates subtle GABAergic hyperpolarization or even depolarization in the adult thalamus. Thalamic reticular nucleus neurons are interconnected by a network of GABAergic synapses (Bazhenov et al., 1999; Sun et al., 2012). Indeed, GABAergic synaptic transmission triggers action potentials in the reticular thalamic nuclei (Bazhenov et al., 1999; Sun et al., 2012), such that GABA-evoked burst firing of GABAergic reticular neurons may result in delayed feedforward inhibition onto the thalamic relay neurons. Also of concern is GABAergic action in the embryonic thalamus because KCC2 but not NKCC1 is expressed in fetuses. Unfortunately, however, there are no reports to date describing electrophysiological studies addressing Cl^−^ homeostasis in fetal neurons. In postnatal rats, spontaneous giant depolarizing potentials have been observed in the thalamic reticular neurons as early as P3 to P8 (Pangratz-Fuehrer et al., 2007). In any case, the above GABAergic functions are consistent with the postnatal expression profiles of KCC2 and NKCC1 (Wang et al., 2002).

The olfactory bulb is formed by projection neurons (mitral and tufted cells) and GABAergic interneurons (granule cells), and it is the first region of the telencephalon to differentiate. The mitral cells first originate around E11; they already express KCC2 at E14.5, and KCC2 expression increases with further development (Li et al., 2002; Stein et al., 2004). Tufted cells appear shortly after mitral cells, followed by granule cells, which are mainly generated around birth and thereafter. The granule cells form a layer underneath the mitral cell layer, and KCC2 expression is observed in this layer at E18.5 in mice (Stein et al., 2004) and at P7 in rats (Wang et al., 2002). NKCC1 is already expressed at birth, and its expression gradually increases until it plateaus at adulthood (Wang et al., 2005). Mitral cells expressing KCC2 are inhibited by GABA in the early postnatal period, whereas granule cells lacking KCC2 are depolarized or even excited by GABA (Wang et al., 2005). After upregulation of KCC2 at P16, granule cells are hyperpolarized and inhibited by GABA. The transient GABA-mediated excitation onto granule cells for a short postnatal period might enhance the inhibitory effects of GABA on mitral cells. Thus, the differential actions of GABA in relay vs. intrinsic neurons play pivotal roles in early postnatal life.

In the cerebellum, at approximately E15, Purkinje cells start to differentiate and axons extend, and around birth, dendrite formation and synaptogenesis occur (Hatten et al., 1997). Granule cells differentiate later, when they start to migrate inward from the external granular layer, and finish their migration into the internal granular cell layer at approximately P3 (Paxinos, 1995). Purkinje cells already express KCC2 at E15.5, and these signals increase between that stage and E18.5. At P3, KCC2 can also be detected in granular cells in mice, and its expression further increases with maturation (Stein et al., 2004). In the rat external granular layer, where immature cells exist, substantial NKCC1 mRNA expression but not KCC2 mRNA is detected on P7 and P14. In contrast, KCC2 mRNA is already expressed in Purkinje cells on P1, after which its expression increases to adult levels, but interestingly, NKCC1 is not expressed at any postnatal age (Mikawa et al., 2002). The mRNA expression of KCC2 and NKCC1 is also observed in postmigratory granule cells in the internal granular layer after P7, after which it increases. The expression of KCC2 and NKCC1 mRNAs reaches adult patterns by P21. Thus, in the rat cerebellum, KCC2 mRNA expression is induced when neurons arrive at their final destinations (Mikawa et al., 2002).

Hippocampal pyramidal cells develop first in the CA3 field and then in CA1, where KCC2 signals can be detected at E15.5 and at E18.5, respectively, in mice (Stein et al., 2004), but a different pattern has been reported in rats (Wang et al., 2002). In the granule cells of the dentate gyrus, KCC2 expression is not detectable at E18. Substantial NKCC1 mRNA expression is found in the hippocampal neuroepithelium at E18. At P1, moderate KCC2 and weak NKCC1 mRNA expression is observed in the pyramidal cell layer of CA1–3. In the dorsal blade of the dentate gyrus, substantial KCC2 but not NKCC1 mRNA expression becomes detectable at P1 but remains weaker than in CA1–3. At P15, KCC2 mRNA expression becomes very strong in the pyramidal cell layer of CA1–3 and in the granular cell layer of the dentate gyrus. The pyramidal cell layer of CA1–3 shows moderate NKCC1 mRNA expression, and the hippocampal granule cell layer exhibits strong NKCC1 mRNA expression. NKCC1 mRNA expression is markedly upregulated postnatally, and its localization shifts from predominantly somatic to dendritic (Marty et al., 2002). The expression patterns are not changed between P15 and P40. Thus, the expression levels of both KCC2 and NKCC1 reach adult levels at approximately P15.

In comparison to other brain structures, the development of Cl^−^ homeostasis is much delayed in the cortex, especially in the hippocampus and neocortex. Ben-Ari et al. (1989) originally showed that in CA3 pyramidal cells in the rat hippocampus, the switch from depolarizing to hyperpolarizing GABAergic signaling occurs at approximately P5. These results are compatible with the emergence of GABAergic IPSPs observed using intracellular recording (Swann et al., 1989). The GABA_A_R-mediated synaptic connections in CA1 neurons are likely to occur at the early stages of postnatal life. This may be parallel with the ontogeny of Cl^−^ homeostasis in the postnatal hippocampus, with KCC2 and NKCC1 mRNA expression levels significantly increased between P1 and P15. The high NKCC1 expression during the first postnatal week is consistent with its role in the depolarizing effects of GABA (Yamada et al., 2004).

In contrast to other parts of the cerebrum, such as the basal ganglia, piriform cortex, and amygdala, it is not possible to detect KCC2 mRNA expression in the neocortex until P0 (Li et al., 2002; Wang et al., 2002). NKCC1 mRNA is already detectable in the ventricular zone (VZ) of the lateral ganglionic eminence at E12.5, and its expression increases significantly in the proliferative zones of the lateral and medial ganglionic eminence by E14.5. At this stage, NKCC1 mRNA expression is also observed in the VZ of the isocortex but not in the cortical plate and subventricular zone (SVZ). Although NKCC1 mRNA expression in the isocortex does not change significantly, its distribution changes from the VZ to the cortical plate (Li et al., 2002; Wang et al., 2002). NKCC1 is expressed in neuronal precursor cells but not in postmitotic migrating or differentiating neurons at approximately E14.5. Interestingly, NKCC1 expression in the VZ disappeared at later stages of embryonic development (E17-P0) corresponding to the cessation of neurogenesis (Caviness et al., 1995). In contrast, NKCC1 mRNA and protein are strongly expressed in the differentiated cortical plate cells.

During early postnatal days in the neocortex, NKCC1 mRNA expression is high and then decreases during development, whereas KCC2 mRNA expression demonstrates an opposite pattern with a marked increase in expression after the first postnatal week (Yamada et al., 2004). NKCC1 mRNA expression is greatest in the VZ, followed by the cortical plate and layer V/VI, while the reverse pattern is apparent for KCC2 mRNA (Shimizu-Okabe et al., 2002). At P15, KCC2 mRNA is expressed strongly in layers II–VI of the cerebral cortex, and expression in layers II–IV is stronger than that in layers V–VI. NKCC1 mRNA is also expressed moderately in layers II–VI of the P15 cerebral cortex. At P40, the expression patterns of KCC2 and NKCC1 mRNAs are the same as those on P15 (Wang et al., 2002).

Consistently, the existence of GABA-containing synaptic contacts occurs at P4 as demonstrated by ultrastructural analyses of developing somatosensory cortex (Owens and Kriegstein, 2002). As mentioned above, the direction of Cl^−^ flux determines the activity of the GABA_A_ receptor channels. Indeed, GABA_A_R-mediated synaptic potentials depolarize postsynaptic cells during the first few postnatal days, suggesting that rapid excitatory synaptic transmission is mediated by GABA_A_Rs in the early-stage neocortex (Owens and Kriegstein, 2002; Qian et al., 2014). The [Cl^−^]_i_ levels in neocortical neurons are consistent with NKCC1 and KCC2 mRNA expression levels; thus, Cl^−^ homeostasis ontogeny could be regulated via differential expression of these Cl^−^ transporters. High [Cl^−^]_i_ levels are considered to be essential for corticogenesis, including neurogenesis, migration and synaptogenesis, and the regional differences may indicate that those cell types are occupying differential developmental stages (Figure 2). The marginal zone exhibits unique Cl^−^ homeostasis and plays important roles in cell migration, lamination, and horizontal synaptic transmission (Qian et al., 2014). In addition, KCC2 is expressed in chronological order of neuronal differentiation (Takayama and Inoue, 2010). The onset of KCC2 localization is concomitant with GABA synapse formation, which suggests that GABA becomes inhibitory soon after GABA synapses are formed.

## Factors Regulating Transcription of Cation-Cl^−^ Co-Transporters

**Figure 3:**
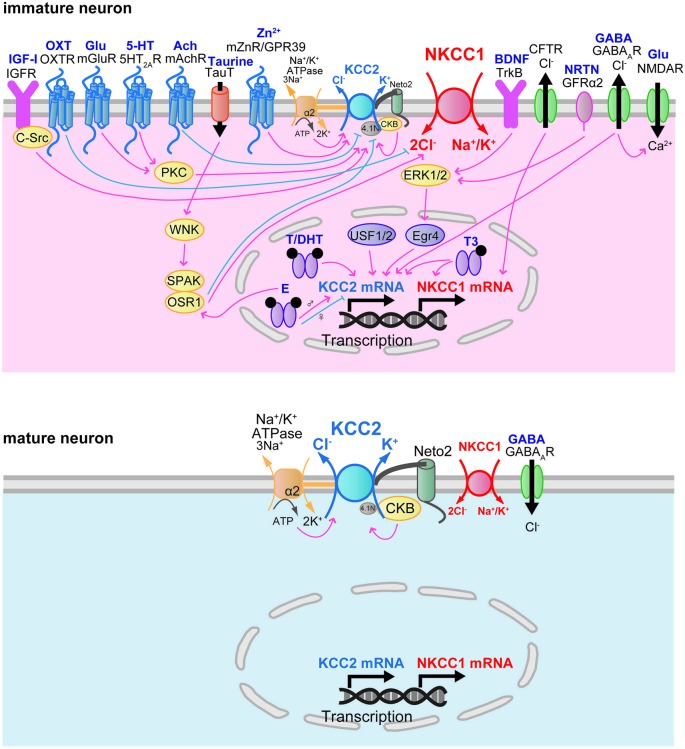
**Developmental changes in factors regulating Cl^−^ transporter transcription and function**.

The developmental increase of factors that regulate NKCC1 and KCC2 gene expression is correlated with the time course of the developmental switch of the GABA_A_R response. The developmental increase in NKCC1 and KCC2 protein expression is modulated by neurotrophic factors (Figure 3). Brain-derived neurotrophic factor (BDNF) exerts a facilitatory effect on the expression of KCC2 mRNA and protein during development. Increased expression of KCC2 mRNA in the hippocampus of BDNF-overexpressing transgenic mice at E18 reduced [Cl^–^]_i_ and then decreased [Ca^2+^]_i_ transients evoked by GABA_A_ receptor activation (Aguado et al., 2003). Conversely, the expression of KCC2 mRNA is decreased in the hippocampus of tyrosine receptor kinase B (TrkB) receptor null mice at P10 (Carmona et al., 2006). This facilitatory effect of BDNF on KCC2 protein expression occurs via extracellular signal-regulated kinase 1/2 (ERK1/2)-dependent upregulation of the transcription factor early growth response 4 (Egr4) and via Egr4-dependent activation of the KCC2b promoter (Ludwig et al., 2011). The trophic factor neurturin (NRTN) could also trigger Egr4 mRNA expression and upregulate KCC2 protein in an ERK1/2-dependent manner in developing neurons (Rivera et al., 2011). NRTN belongs to the GDNF family of neurotrophic factors and binds to the GPI-anchored receptor GDNF family receptor 2 (GRFα2).

These results suggest that Egr4 is an important component in the mechanism for trophic factor-mediated upregulation of KCC2 protein in developing neurons. However, a contradictory result using BDNF null mice was reported, showing that a complete absence of BDNF did not affect the developmental upregulation of KCC2 protein and its function in the hippocampus (Puskarjov et al., 2015). BDNF has also been reported to regulate NKCC1 protein expression. NKCC1 protein expression was upregulated in the rat hippocampus of pilocarpine-induced temporal lobe epilepsy (Eftekhari et al., 2014). This upregulation of NKCC1 expression was decreased by BDNF application (Eftekhari et al., 2014). In the adult brain, BDNF mRNA and protein were increased in the hippocampus by seizure (Binder and Scharfman, 2004). Kindling-induced seizures (Rivera et al., 2002) and interictal-like activity (Rivera et al., 2004) activated BDNF-TrkB signaling and downregulated KCC2 protein conversely to immature brain. BDNF and TrkB are widely expressed in the brain at both the mRNA and protein levels (Conner et al., 1997; Yan et al., 1997a,b). The expression of BDNF mRNA increases in the whole brain during development (Maisonpierre et al., 1990). In the hippocampus, the expression of BDNF mRNA is low at E17, increased at P0, and high in the adult (Maisonpierre et al., 1990). In the cerebellum, BDNF mRNA expression begins to increase at P11 and reaches a high level in the adult (Maisonpierre et al., 1990). In the neocortex, BDNF mRNA expression increases during development (Maisonpierre et al., 1990). In the spinal cord, BDNF expression reaches detectable levels at E12, peaks at P0, and then decreases in the adult (Maisonpierre et al., 1990). The mRNA expression of NRTN in the hippocampus peaks at E14, then decreases during E16-P0, and increases again by P5 (Lenhard and Suter-Crazzolara, 1998). At P4–14, GRFα2 mRNA is highly expressed in the granule cell layer of the olfactory bulb, in the pyramidal layer of the hippocampus, layers IV/VI of the cortex, and in the cerebellum (Burazin and Gundlach, 1999).

Another transcription factor that has been identified as a potent regulator of KCC2 expression is upstream stimulating factor 1 (USF1) as well as USF2. The regulatory influence of the ubiquitously expressed proteins USF1 and USF2 focuses on the major E-box binding complex of the KCC2b promoter, and the interaction of USF proteins through the E-box element contributes to the activation of KCC2b gene expression in cultured cortical neurons (Markkanen et al., 2008). Mice deficient in USF1 or USF2 showed spontaneous epileptic seizures, suggesting that USF1 and USF2 are important for normal brain function (Sirito et al., 1998). USF1 and USF2 have been reported to play a critical role in mediating activity-dependent gene expression in neurons (Tabuchi et al., 2002; Chen et al., 2003; Steiger et al., 2004).

The developmental change in the expression of KCC2 and NKCC1 is modulated by humoral factors. Triiodothyronine (T3) increased the expression of the NKCC1 protein in cultured cortical neurons at 14 and 21 days *in vitro* (DIV), while age-dependent change in NKCC1 expression is not influenced (Westerholz et al., 2013). T3 also enhanced the expression of KCC2 protein at 14 and 21 DIV and accelerated the developmental shift of GABA action from depolarizing to hyperpolarizing (Westerholz et al., 2013). Consistent with this study, in rats with hypothyroidism, the switching of GABA action in lateral superior olive neurons was delayed by 7 days (Friauf et al., 2008), and decreases in the expression of KCC2 mRNA and protein in the hippocampus were observed at P10 and P15 (Sawano et al., 2013). The expression of the thyroid hormone receptor in the brain increases during the postnatal period (Perez-Castillo et al., 1985).

Oxytocin (OXT) has been reported to modulate NKCC1 function during development. Shortly before delivery, OXT triggers the transient switch of the GABA response from excitatory to inhibitory by inhibiting NKCC1 activity in the hippocampal CA3 neurons (Tyzio et al., 2006). Parturition is initiated by a massive increase in oxytocin release (Gimpl and Fahrenholz, 2001), and maternal oxytocin crosses the placenta to reach the fetus. High expression of the oxytocin receptor (OXTR) was observed in the fetal hippocampus in the perinatal period (Tyzio et al., 2006). In immature cultured hippocampal neurons (2 DIV), oxytocin increased cellular viability both immediately after oxygen-glucose deprivation (OGD) and after 6 h of reoxygenation (Ceanga et al., 2010). Application of the NKCC1 inhibitor bumetanide during OGD demonstrated a neuroprotective effect after reoxygenation comparable to that of oxytocin, indicating that oxytocin may inhibit NKCC1 function.

Oxytocin appears to represent a key signal that relates chronic hyperosmotic stress to the depolarizing switch of the GABA response in adult rats (Kim et al., 2011). Chronic hyperosmotic stress converted the GABA response from inhibitory to excitatory in rat hypothalamic magnocellular neurosecretory cells. This conversion was associated with an increase in NKCC1 protein expression, and blocking OXTRs prevented the shift in the GABA response. This report suggests that the switch in GABA action contributes to the increased secretion of arginine–vasopressin and oxytocin. Gonadal hormones have also been suggested to regulate the expression of KCC2. KCC2 exhibits sexually dimorphic expression in neurons of the substantia nigra pars reticulata (SNR). In male rats, SNR neurons at P15 showed low KCC2 mRNA expression and depolarization in response to GABA (Galanopoulou and Moshé, 2003; Galanopoulou et al., 2003). Acute subcutaneous administration of a GABA_A_ receptor agonist, muscimol, upregulated KCC2 mRNA, whereas an L-type voltage-gated calcium channel blocker, nifedipine, downregulated KCC2 mRNA (Galanopoulou et al., 2003). In female rats, however, muscimol downregulated KCC2 mRNA, whereas nifedipine had no effect. Estradiol downregulates KCC2 mRNA in males but not in females. This downregulation of KCC2 mRNA is prevented by pretreatment with nifedipine or bicuculline. Furthermore, estradiol decreases the expression of the phosphorylated form of the transcription factor cAMP responsive element binding protein (phosphoCREB) in dopamine neurons of the SNR in males but increases its expression in females (Galanopoulou, 2006).

These data suggest that GABA_A_ receptor activation and estradiol promote the sexual differentiation of KCC2 expression in the SNR via phosphoCREB-mediated gene transcription. Subcutaneous administration of testosterone and dihydrotestosterone (DHT) upregulated KCC2 mRNA in both sexes (Galanopoulou and Moshé, 2003). The SNR shows greater immunoreactivity for both estrogen receptor α and androgen receptor in males than in females at P15, whereas the immunoreactivity for estrogen receptor β does not differ (Ravizza et al., 2002).

Although a later study showed controversial results (Ludwig et al., 2003), the depolarizing action of GABA itself had previously been suggested to facilitate the upregulation of KCC2 through Ca^2+^-dependent transcription in cultured hippocampal neurons (Ganguly et al., 2001). The developmental increase in spontaneous GABA release is tightly correlated with the time course of the developmental switch of the GABA response.

Cystic fibrosis transmembrane conductance regulator (CFTR) is a cAMP-regulated Cl^−^ channel that plays a role in neonatal rat spinal motoneurons. The gene activities of the NKCC1 and CFTR were positively correlated and increased between P1 and P8 in motoneurons of the rat spinal cord (Ostroumov et al., 2011). A selective CFTR blocker, diphenylamine-2,2′-dicarboxylic acid, produced a negative shift in E_GABA_/E_glycine_, indicating that CFTR induces depolarizing GABA/glycine-mediated synaptic events (Ostroumov et al., 2011).

## Factors Regulating Post-Translational Modification of Cation-Cl^−^ Co-Transporters

The expression and activity of factors that regulate the activity of NKCC1 and KCC2 closely parallel the functional expression of NKCC1 and KCC2 in the developing brain. Khirug et al. (2010) suggested that in the neonatal brain, overall KCC2 protein expression levels appear to be high enough to cause a rapid activation of KCC2 and consequently to induce a hyperpolarized shift of E_GABA_ similar to that in the adult brain. Neonatal seizure episodes induced by kainate injection during P5–7 resulted in a rapid and pronounced increase in KCC2-mediated Cl^−^ extrusion from rat hippocampal CA1 neurons, leading to a hyperpolarizing shift of E_GABA_ (Khirug et al., 2010). Although the total protein expression of KCC2 was not changed, the increase in Cl^−^ extrusion efficacy was associated with the increase in KCC2 in the plasma membrane. The developmental activation of KCC2 function is mediated by post-translational modifications of KCC2 such as phosphorylation or dephosphorylation events (Figure 3). Numerous putative KCC2 phosphorylation sites are reported to contribute to the functional regulation of KCC2 (Medina et al., 2014). The broad spectrum kinase inhibitor staurosporine rapidly upregulates KCC2 activity in immature cultured hippocampal neurons (5–10 DIV; Khirug et al., 2005).

Lee et al. (2007) identified serine 940 in the C-terminal domain of KCC2 as a major site for protein kinase C (PKC), and phosphorylation of serine 940 decreased the rate of internalization of KCC2 from the cell membrane. Activation of 5-hydroxytryptamine (5-HT) type 2A receptors and group I metabotropic glutamate receptors (mGluR1s) has been reported to modulate KCC2 function through PKC-dependent mechanisms. Activation of 5-hydroxytryptamine type 2A (5-HT_2A_) receptors increased the cell membrane expression of KCC2 and upregulated KCC2 function through a PKC-dependent signaling pathway in spinal motor neurons during P5–7, and this activation reduced spinal cord injury-induced spasticity (Bos et al., 2013). In CA3 pyramidal neurons at P15–22, application of an mGluR1 agonist causes a hyperpolarizing shift of E_GABA_, and an mGluR1 antagonist produces a depolarizing shift of E_GABA_ (Banke and Gegelashvili, 2008). A PKC activator mimicked the effects of the mGluR1 agonist, and a Ca^2+^-dependent PKC inhibitor mimicked the effects of the mGluR1 antagonist, indicating that tonic activation of mGluR1s regulates inhibitory synaptic strength via activation of a PKC-dependent pathway to change the KCC2 activity, thereby changing the [Cl^−^]_i_. The mRNA expression of PKCα, β, and γ is weak at P0, increases at P7, and then decreases at P21 in the hippocampus, cortex, and cerebellum (Minami et al., 2000). Threonine (Thr)-906 and Thr-1007 in KCC2 are reported to be phosphorylated in perinatal mouse brain and dephosphorylated during the course of postnatal development (Rinehart et al., 2009). The substitution of Thr-906 and Thr-1007 with alanine facilitated KCC2 activity in HEK cells (Rinehart et al., 2009) and in E18.5 rat cortical neurons (Inoue et al., 2012), suggesting that KCC2 activity is inhibited by phosphorylation of Thr-906 and Thr-1007 in perinatal brain. This developmental change in phosphorylation is modulated by taurine via with-no-lysine protein kinase 1 (WNK1) and the downstream SPAK/oxidative stress responsive kinase-1 (OSR1) pathway (Inoue et al., 2012). WNK, a serine-threonine kinase, comprises four members (WNK1–4). KCC2 and NKCC1 activities are reciprocally regulated via phosphorylation of WNK and of the downstream SPAK/OSR1 (Alessi et al., 2014). Expression of dominant negative WNK1 and genetic silencing of WNK1 using shRNA increased KCC2 activity in the hippocampal neurons (6–7 DIV; Friedel et al., 2015). WNK3, which is highly expressed in the brain, activates NKCC1 via phosphorylation of the N-terminal threonine residues, whereas dephosphorylation of these residues by protein phosphatase 1 inhibits NKCC1 activity (Kahle et al., 2005). In contrast, WNK3 inhibits KCC2 activity via the phosphorylation of C-terminal threonine residues (Kahle et al., 2005, 2013). The expression of WNK3 mRNA in human hippocampus and neocortex is developmentally regulated in a reciprocal fashion relative to that of KCC2 (Kahle et al., 2013), suggesting that WNK3 activity might contribute to the switching of GABA action from excitatory to inhibitory during development by modulating KCC2 activity. WNK2 is exclusively expressed in the brain starting from the embryonic period and promotes Cl^−^ uptake by activating NKCC1 and inhibiting KCC2 (Rinehart et al., 2011). Therefore, the reciprocal actions of WNK on NKCC1 and KCC2 might be important for modulating [Cl^−^]_i_ and plasticity in the GABA response by altering NKCC1 and KCC2 activity.

Taurine increases phosphorylation of serine 382 in WNK1, serine 373 in SPAK, and serine 325 in OSR1 (Inoue et al., 2012). Phosphorylation of WNK1 is not changed at P1 and P7 (Inoue et al., 2012) and is decreased at P14 (Friedel et al., 2015). The phosphorylation of SPAK decreases at P1 and P7 compared to that of E18.5, suggesting that dephosphorylation of SPAK facilitates KCC2 activity. Taurine has been reported to play an important role in the developing brain (Sturman, 1988). Taurine is abundant in the cortex, cerebellum, and olfactory bulb in P4 rats (Shimada et al., 1984) and activates glycine receptors in the cortex (Flint et al., 1998).

NKCC1 activity is modulated by gonadal hormones in the neonatal hypothalamus. Estradiol increased the amplitude of the Ca^2+^ increase induced by muscimol and extended the developmental time course of excitatory responses to GABA (Perrot-Sinal et al., 2001). Estradiol enhanced NKCC1 activity via increased phosphorylation of NKCC1 at Thr-212 and Thr-217 (Perrot-Sinal et al., 2007). Estradiol treatment significantly increased protein levels of SPAK/OSR1, and knockdown of SPAK and OSR1 prevented estradiol-mediated enhancement of NKCC1 phosphorylation (Nugent et al., 2012), indicating that estradiol activates the SPAK/OSR1 pathway and consequently increases NKCC1 phosphorylation and enhances NKCC1 activity.

The activity of tyrosine kinases may also influence the developmental change in KCC2 activity. Kelsch et al. (2001) reported that cultured hippocampal neurons initially expressed an inactive form of the KCC2 protein (23 DIV), which became activated during subsequent maturation of the neurons (35–44 DIV). This process was accelerated by insulin and insulin-like growth factor-1 (IGF-1) in conjunction with a cytosolic protein tyrosine kinase, C-Src tyrosine kinase (c-Src), and could be deactivated by genistein or lavendustin A, which are membrane-permeable protein tyrosine kinase inhibitors, indicating that the development of KCC2 activity requires cooperation of a growth factor with tyrosine kinase-dependent phosphorylation (Kelsch et al., 2001). Tyrosine phosphorylation of KCC2 was enhanced by the activation of muscarinic acetylcholine receptors (mAChRs) in cultured hippocampal neurons (21 DIV; Lee et al., 2010). Contradictory results were reported by Stein et al., indicating that phosphorylated KCC2 protein was already present early in development when the functional GABA switch had not yet occurred; they therefore concluded that tyrosine phosphorylation may be less important than the transcriptional upregulation of KCC2 (Stein et al., 2004).

IGF-1 signaling through the IGF-1 receptor is crucial for normal brain development and stimulates neuron progenitor proliferation, neuron survival, neurite outgrowth, and synaptogenesis (Russo et al., 2005; Liu et al., 2014). IGF-1 is widely expressed in the CNS, and its expression temporally and spatially correlates with brain development (Bondy, 1991). The IGF-1 receptor is highly expressed in the developing hippocampus (Kar et al., 1993). In rodent brains, IGF-1 expression increases rapidly during late prenatal and early postnatal development and peaks during the first postnatal week (Rotwein et al., 1988; Popken et al., 2005). The IGF receptors are expressed widely in the brain, and their expression pattern is also developmentally regulated (Liu et al., 2014).

Recent studies have demonstrated that zinc controls KCC2 activity via a postsynaptic metabotropic zinc receptor/G protein-linked receptor 39 (mZnR/GPR39; Besser et al., 2009; Chorin et al., 2011). The physiological activation of mZnR/GPR39 caused an increase in the surface expression and activity of KCC2 in CA3 neurons of P12–15 mice (Chorin et al., 2011). The effects of Zn^2+^ on KCC2 activity were also shown in cultured cortical neurons from wild-type mice at 18–25 DIV but not in cortical neurons obtained from mZnR/GPR39^−/−^ mice (Saadi et al., 2012). The hippocampal slices obtained from the mZnR/GPR39^−/−^ mice lacked the kainate-induced synaptic Zn^2+^ release and upregulation of KCC2 transport activity that were observed in the tissue obtained from their wild-type littermates (Gilad et al., 2015). Kainate-dependent upregulation of KCC2 requires mZnR/GPR39 activation of the Gq-mediated signaling pathways that lead to ERK activation. The levels of both synaptic Zn^2+^ and KCC2 are developmentally upregulated. During the postnatal period, synaptic Zn^2+^ accumulation and KCC2 expression reach levels similar to those in adult brain (Slomianka and Geneser, 1997). The zinc transporter 1 (ZnT-1), which is present in areas rich in synaptic zinc, is expressed from the first postnatal week in cortex, hippocampus, olfactory bulb (Nitzan et al., 2002). In the cerebellum, the expression of ZnT-1 in purkinje cells is increased during the second postnatal week.

## Factors Regulating Localization and/or Assembly of Cation-Cl^−^ Co-Transporters

It has been reported that monomeric KCC2 is transport-inactive and that age-dependent oligomerization activates KCC2, thereby decreasing [Cl^−^]_i_ (Blaesse et al., 2006)_._ This change may cause the developmental shift in GABA_A_R action from depolarization to hyperpolarization. The single-pass transmembrane protein neuropilin and tolloid like-2 (Neto2) is predominantly associated with the active oligomeric form of KCC2 and maintains normal efficient KCC2-mediated Cl^−^ extrusion (Ivakine et al., 2013). Loss of the interaction between Neto2 and KCC2 reduces KCC2 activity, leading to decreases in synaptic inhibition in the hippocampal neurons. Neto2 mRNA expression is first detected at E15 and then increases between P5 and 21 in the neocortex, hippocampus, cerebellum, and thalamus (Michishita et al., 2004). This expression pattern closely parallels the expression of KCC2 during the same developmental period.

The interaction between cytoskeleton-associated protein 4.1N and KCC2 play an important role in the morphological maturation of cortical neurons (Li et al., 2007). KCC2 is a key molecule in the maturation of dendritic spines and functional excitatory synapses. The maturation of dendritic spines does not occur in the absence of KCC2 expression. Surprisingly, this morphogenic role of KCC2 does not require its Cl^−^ transport activity. Both KCC2 and 4.1N are abundant in neurons during early development at the time of synaptogenesis, and their expression is well correlated with the maturation of excitatory synapses (Walensky et al., 1999; Ludwig et al., 2003). 4.1N mRNA is widely expressed in the CNS; it is detected at the earliest stage of postmitotic differentiation and is abundant in postmitotic neurons in the cerebral cortex from E11.5 to P21 (Walensky et al., 1999). At the postsynaptic densities of climbing fibers on purkinje neurons, the protein expression of 4.1N is high at E19, gradually decreases from E19 to P10, and cannot be detected at P13 (Douyard et al., 2007).

## Factors Regulating Electrochemical Driving Force of Cation-Cl^−^ Co-Transporters

In addition to the factors involved in protein-protein interactions mentioned above, factors regulating the electrochemical driving force of Cl^−^ transporters also contribute to the development of Cl^−^ homeostasis. Both the Na^+^-K^+^ ATPase α2 subunit and brain-type creatine kinase (CKB) directly bind to KCC2 and are essential for the proper activity of KCC2 (Ikeda et al., 2004; Inoue et al., 2004, 2006). CKB transfers high-energy phosphate from phospho-creatine to ADP to rapidly generate ATP. Although direct binding between the Na^+^-K^+^ ATPase α2 subunit and CKB has not been reported, hypothetically, via binding separately to KCC2, they may be functionally coupled as a generator and acceptor of ATP (Blum et al., 1991). Then, Na^+^-K^+^ ATPase hydrolyzes the given ATP and generates robust Na^+^ and K^+^ electrochemical gradients, allowing KCC2 to utilize the K^+^ electrochemical gradient as a driving force to export Cl^−^. Therefore, in the subcellular microenvironment, a proper increase in KCC2 function may require not only the developmental increase in KCC2 protein but also concomitant increases in CKB and Na^+^-K^+^ ATPase.

The Na^+^-K^+^ ATPase consists of α and β subunits. Four α isoforms (α1, α2, α3, and α4) have been identified in mammals. Each isoform exhibits a unique tissue distribution and expression pattern during development. The α1 isoform is expressed ubiquitously and is indispensable for early embryonic development. The α2 and α3 isoforms are expressed specifically and abundantly in skeletal muscle, heart, and brain. The α1 isoform is distributed throughout the plasma membrane, whereas α2 and α3 localize in more specific areas, including the postsynaptic area (Fukuda and Prince, 1992; Dolapchieva, 1996). Interestingly, only the α2 isoform functionally interacts with KCC2 (Ikeda et al., 2004). The mRNA of the α2 isoform is distributed throughout the brain at E9.5 and becomes expressed in more restricted regions (Herrera et al., 1994). At birth, the α2 isoform is present in neuronal cell bodies at all levels in the brain, as well as in glial cells (Moseley et al., 2003). In the rat, the dynamics of the Na^+^-K^+^-ATPase including the α2 subunit of the synaptic regions during postnatal development of the hippocampus and cerebral cortex have been reported. In the cortex, the postsynaptic density is lacking in Na^+^-K^+^-ATPase at P10, but some postsynaptic densities express it at P15. The specific and uniform distribution of Na^+^-K^+^-ATPase in the postsynaptic densities is established at approximately P60 (Dolapchieva, 1996). In the hippocampus, Na^+^ pump activity is very low in the first postnatal week and develops gradually over the next 4 weeks of life. The Na^+^-K^+^-ATPase weakly surrounds cell bodies and dendrites at P7 and then develops a characteristic focal localization. Thus, the Na^+^-K^+^-ATPase levels in hippocampal CA1 pyramidal neurons are insufficient to allow substantial Na^+^ pump activity in the first postnatal week. The adult functional levels of Na^+^-K^+^ pump activity are gradually attained over subsequent weeks (Fukuda and Prince, 1992). Thus the developmental changes in Na^+^-pump activity are associated with the establishment of the postsynaptic activity in both the hippocampus and cortex.

There are four isoenzymes of creatine kinase (CK): one brain (CKB), one muscle (CKM), and two mitochondrial CKs (Wyss and Kaddurah-Daouk, 2000). CKB and CKM are functionally active as dimers: BB (the predominant form in brain), MM (in skeletal muscle) and MB (in cardiac muscle). Of these, only CKB interacts with KCC2; amino acids 1048–1114 of KCC2 and 273–381 of CKB are important for the interaction (Inoue et al., 2004). Notably, CKB does not contribute to NKCC function (Inoue et al., 2006). In rat brains, CKB mRNA is detected as early as E16, and its levels gradually increase during the first 2 weeks after birth, reaching a peak of approximately four times that observed at E16, correlating with increased BB isoenzyme activity and more complex nervous system activity (Trask and Billadello, 1990). Manos et al. (1991) observed a highly similar result, with cytosolic CKB increased 4–5-fold from P0 to P40 in developing rat brain, with the most rapid increase at approximately P10. The postnatal increase in CKB activity may be attributed to neuronal energy requirements for synaptogenesis.

At early stages of development (i.e., the first postnatal week), when inhibitory inputs are absent or less effective in hippocampus and neocortex (Luhmann and Prince, 1991; Fukuda and Prince, 1992; Fukuda et al., 1993), the GABA_A_ receptor mediates depolarization due to high intracellular Cl^−^ (Fukuda et al., 1998; Yamada et al., 2004). Thus, the development of KCC2-bound proteins, including CKB and the α2 subunit of Na^+^-K^+^-ATPase, is well correlated with that of KCC2, such that the subcellular microenvironment may underlie the ontogeny of inhibitory GABAergic function.

## Conclusion

In conclusion, the development of Cl^−^ homeostasis and the switch from high to low [Cl^−^]_i_ is accompanied by regionally differential KCC2 upregulation with or without concomitant NKCC1 downregulation. The timing of the depolarization–hyperpolarization switch of GABA action is also accompanied by maturation of the brain, including synaptogenesis. Posttranslational mechanisms such as protein-protein interactions and phosphorylation and the subcellular microenvironment for the electrochemical driving force also show synergistic development with that of KCC2, indicating the purposeful spatiotemporal development of Cl^−^ transporters and their regulators. Importantly, in adulthood, most of the transcriptional and posttranslational modulations of Cl^−^ transporters become inactive but can be activated by certain traumatic events.

## Conflict of Interest Statement

The authors declare that the research was conducted in the absence of any commercial or financial relationships that could be construed as a potential conflict of interest.

## Abbreviations

Ach: acetylcholine
BDNF: brain-derived neurotrophic factor
CFTR: cystic fibrosis transmembrane conductance regulator
CKB: brain-type creatine kinase
C-Src: C-Src tyrosine kinase
DHT: dihydrotestosterone
E: estrogen
Egr4: early growth response transcription factor 4
ERK1/2: extracellular signal-regulated kinase 1/2
GRFα2: GDNF family receptor 2
Glu: glutamate
mAchR: muscarinic acetylcholine receptor
mGluR: group I metabotropic glutamate receptor
IGF-I: insulin-like growth factor-1
IGFR: insulin-like growth factor receptor
Neto2: neuropilin and tolloid like-2
NMDAR: N-methyl-D-aspartate receptor
NRTN: neurturin
OSR1: oxidative stress responsive kinase-1
OXT: oxytocin
OXTR: oxytocin receptor
PKC: protein kinase C
SPAK: STE20/SPS1-related proline/alanine-rich kinase
T: testosterone
T3: triiodothyronine
TauT: taurine transporter
TrkB: tyrosine kinase B receptor
USF1/2: upstream stimulating factors 1 and 2
WNK: with-no-lysine protein kinase
Zn^2+^: zinc
mZnR/GPR39: metabotropic zinc receptor/G protein-linked receptor 39
5-HT: 5-hydroxytryptamine
5-HT_2A_R: 5-hydroxytryptamine type 2A receptor.
